# Supplementary material for: Causal interplay between lactose intolerance and gut microbiota: a combined bidirectional Mendelian randomization and in vivo validation study
Source: Front Nutr. 2026 Jun 1;13:1803337. doi: 10.3389/fnut.2026.1803337 (PMC13265576; doi:10.3389/fnut.2026.1803337)
Supplement: Supplementary file 1 [file Data_Sheet_1.zip › supplementary materials/Forward/leave one out cross validation/ebi-a-GCST90027513.finngen_R12_E4_LACTONAS.pdf]

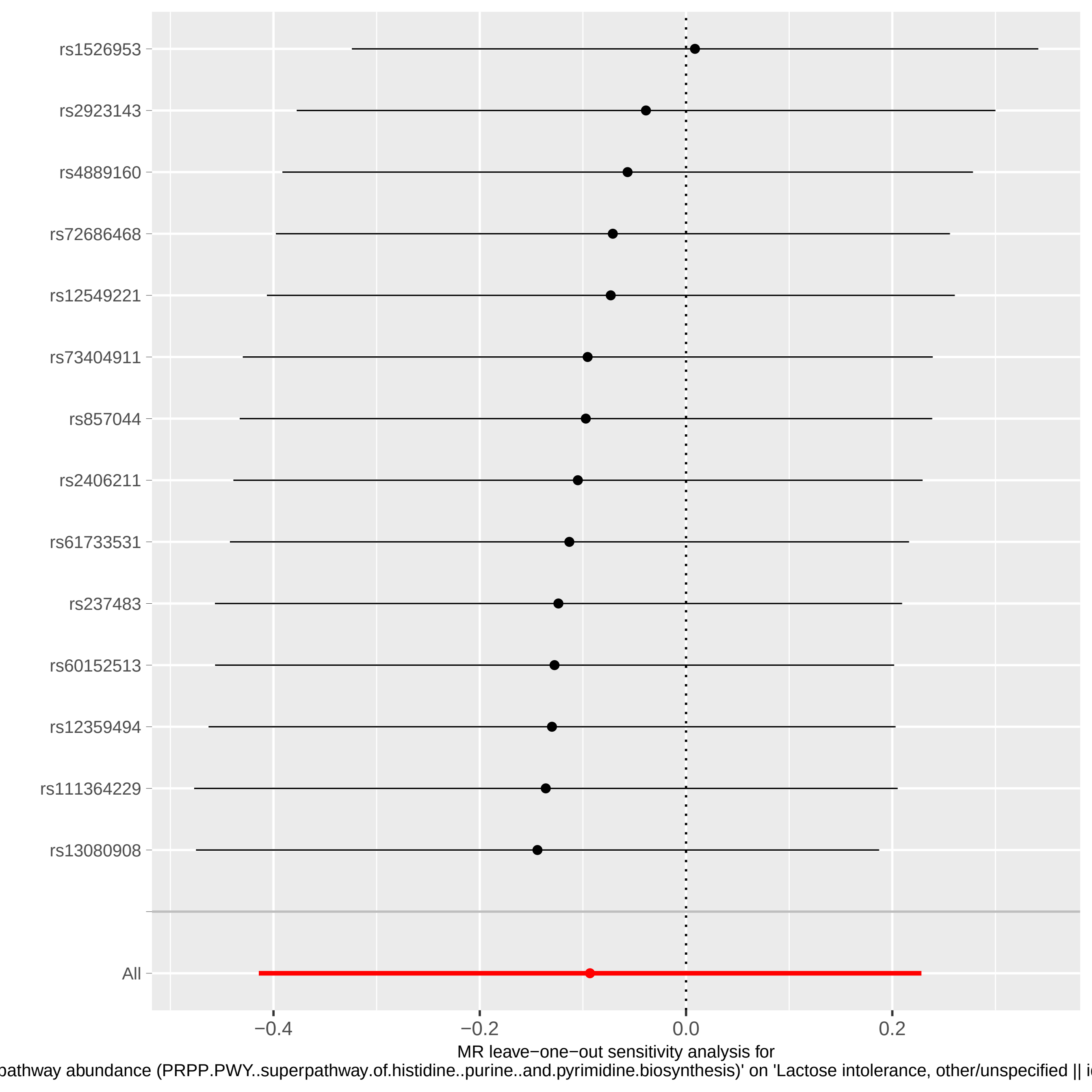

rs1526953
rs2923143
rs4889160
rs72686468
rs12549221
rs73404911
rs857044
rs2406211
rs61733531
rs237483
rs60152513
rs12359494
rs111364229
rs13080908
All
−0.4
−0.2
0.0
0.2
MR leave−one−out sensitivity analysis for
pathway abundance (PRPP.PWY..superpathway.of.histidine..purine..and.pyrimidine.biosynthesis)' on 'Lactose intolerance, other/unspecified || i
